# Treatment of Asthma

**Published:** 1872-11

**Authors:** 


### "Treatment of Asthma."

The treatment of asthma, as Dr. Williams observes, must vary much in its simplicity and success according to the unity or complication of the disease. Against the bronchial spasm we have remedies which are pretty effectual in most cases. Belladonna and stramonium rarely fail to relieve the bronchial spasm; and in transient cases, where this is the only element, they may suffice to cure the disease. The extracts are the most reliable preparations, and may be given in doses of from a quarter of a grain to half a grain every three, four or six hours, while the tendency to spasm lasts. The dryness of the throat, which both these drugs often cause, may be counteracted by frequently sipping linseed-tea or barley-water. Sometimes, however, this dryness is useful in moderating the catarrhal flux which may follow the spasm; but in most cases there exists something more than the mere spasm, and therefore we commonly have to give these antispasmodics in combination with other remedies. Thus, often there is an inflammatory cold, calling for the addition of the salines and counter-irritation; and this may amount to bronchitis, requiring the aid of small doses of tartarized antimony. In chronic cases, when the attacks have recurred frequently or lasted long, there is no combination more beneficial than that of iodide of potassium, in two or three-grain doses, and ten or fifteen grains of bicarbonate of potass, with the stramonium or belladonna. Dr. Williams believes that he speaks within bounds when he says that with a combination of this kind he has cured or greatly relieved hundreds of cases of asthma. The diuretic or eliminative action of these medicines may be advantageously increased in some cases by the addition of squill, colchicum, or tincture of cantharides, particularly where there are indications of gout or of diseases of the skin. On a similar principle in chronic cases certain mineral waters are sometimes useful, particularly those of Eauxbonnes and Cauterets in the Pyrenees, Vichy, and Ems. There are several other remedies for asthma in common use, generally much inferior in efficacy to the preceding, but occasionally useful as subsidiary aids, and sometimes they are our chief resources where those disagree. Such is the etheral tincture of lobelia, which in doses of from twenty to sixty drops he has known in a few instances to be quite successful; but more frequently it has failed, and sometimes caused much nausea and discomfort. Indian hemp, in doses of a grain of the extract, gave signal relief in two cases; but in others it quite failed, and sometimes caused distressing disturbance of the brain and head. Smoking cigarrettes of stramonium, or of the *Datura tatula*, inhaling chloroform (which for safety should be mixed with sulphuric ether and alcohol,) and breathing the fumes of burning niter-paper, are expedients which often give relief in individual cases; and although this relief is less complete and permanent than that following the use of the remedies first recommended, yet they may be useful when these fail, and being prompt in operation, may be employed to ward off slight attacks, where stronger agents are not required, or before the latter can be brought into effective operation. In some cases change of air succeeds wonderfully, and this not always when the change has been of the most salubrious character. In fact the caprices of asthma with regard to air are very curious, and can hardly be accounted for. In most instances, however, a dry atmosphere agrees better than a damp one, and the air of a large town better than that of the country, especially if this be low and damp. *American Practitioner.*

